# Spatially selective excitation of spin dynamics in magneto-photonic crystals by spectrally tunable ultrashort laser pulses

**DOI:** 10.1515/nanoph-2022-0233

**Published:** 2022-05-31

**Authors:** Daria A. Sylgacheva, Nikolai E. Khokhlov, Petr I. Gerevenkov, Iaroslav A. Filatov, Mikhail A. Kozhaev, Igor V. Savochkin, Andrey N. Kalish, Alexandra M. Kalashnikova, Vladimir I. Belotelov

**Affiliations:** Faculty of Physics, M. V. Lomonosov Moscow State University, 1 bld. 2 Leninskie Gory, 119991, Moscow, Russia; Russian Quantum Center, 30 bld. 1 Bolshoy Bulvar, Skolkovo IC, 121205, Moscow, Russia; Research Center for Functional Materials and Nanotechnologies, V. I. Vernadsky Crimean Federal University, 12 Studencheskaya, 295007, Simferopol, Russia; Ioffe Institute, 26 Politekhnicheskaya, 194021, St. Petersburg, Russia; NTI Center for Quantum Communications, National University of Science and Technology MISiS, 4 Leninsky prospekt, 119049, Moscow, Russia

**Keywords:** magneto-optics, magneto-photonic crystals (MPC), nanophotonics, spin dynamics, ultrafast magnetization

## Abstract

In this work, we tackle the problem of the spatially selective optical excitation of spin dynamics in structures with multiple magnetic layers. The 120 fs circularly polarized laser pulses were used to launch magnetization precession in an all-dielectric magneto-photonic crystals (MPC) formed by magnetic layers sandwiched between and inside two magnetic Bragg mirrors. Optical pump-probe experiments reveal magnetization precession triggered via ultrafast inverse Faraday effect with an amplitude strongly dependent on the pump central wavelength: maxima of the amplitude are achieved for the wavelength tuned at the cavity resonance and at the edge of the photonic bandgap. The optical impact on the spins caused by the inverse Faraday effect and spectrum of this effect are found to correlate mostly to the direct Faraday effect. We show that even though the pump laser pulses propagate along the whole structure tuning their wavelength allows localization of a larger spin precession either in the cavity layer or in the Bragg mirror layers selectively. The results pave the way to the ultrafast optical control of magnetization dynamics at a sub-wavelength scale that is vital for modern magneto-photonics and magnonics.

## 1 Introduction

It was demonstrated in the first decade of the century that circularly polarized femtosecond laser pulses can trigger fast coherent spin dynamics in a magnetic medium via the ultrafast inverse Faraday effect (IFE) [1]. IFE is described by a pulse of effective magnetic field that appears inside a magnetic medium during the propagation of a laser pulse. If the induced effective magnetic field is not co-directed with the spins, it launches spin dynamics and spin waves, particularly [2]. Apart from IFE, there are other opto-magnetic and photo-magnetic effects providing control of magnetic states by light [3–5]. All these phenomena open up ample opportunities for magnonics and spintronics [6], since they advance conventional electronic approaches in contactless character, local impact within the micron area together with possibility to control the spin waves parameters [7–13].

So far, the main progress in ultrafast laser excitation of spin dynamics via opto-magnetic effects has been achieved for homogeneous films and bulk crystals. However, the nanophotonic approach offers new benefits. For instance, it has been demonstrated recently that a special nanostructuring of a magnetic medium or deposition of a nonmagnetic nanostructure on a smooth magnetic film provides a significant magneto-optical interaction enhancement and therefore boosts magneto-optical effects [14–22]. On the other hand, nanophotonic approaches are highly useful for tailoring inverse magneto-optical impact [23–31]. A decade ago, a plasmonic structure containing periodically perforated gratings of subwavelength holes on an iron garnet film was proposed to enhance the IFE [24, 32, 33]. Those results paved a way for further theoretical studies [25] and for subsequent experiments [26–31]. For instance, the deposition of gold gratings on magneto-photonic crystals (MPC) enables the excitation of additional resonances in the optical spectra of the structure, the enhancement of magneto-optical [34–36], and hence, potentially, of inverse magneto-optical effects.

Unlike metallic nanophotonic structures the all-dielectric ones [37–41] are more tolerant to high laser pulse intensities required for IFE while also providing significant advantages in enhancement of magneto-optical and opto-magnetic interactions with respect to smooth films and bulk media. As it was demonstrated in e.g., [30], nanopatterning of a garnet film by a 1D grating of trenches allows localization of light in spots with sizes of tens of nanometers and thus launching the exchange standing spin waves with wavelengths smaller than light diffraction limit. The important advantage of nanophotonic structures is that they allow three-dimensional localization of light by tailoring different optical modes which might be advantageous for 3D opto-magnonic applications. For example, a wavelength-selective excitation of perpendicular standing spin waves by guided modes in a magnetic dielectric film has been proposed recently [42]. In this respect opto-magnonic nanocavities are quite promising. Thus, it was shown theoretically that the perpendicular standing spin waves resonating in a structure consisting of thin magnetic dielectric films sandwiched in between the dielectric Bragg mirrors, interact efficiently with light leading to a strong magneto-optical interaction beyond the linear regime and providing enhanced modulation of light through multimagnon absorption and emission processes [43, 44]. The other theoretical work suggested that non-uniform distribution of the optical power within a magnetic layer inside the opto-magnonic cavity allows one to launch perpendicular standing spin waves of different orders by femtosecond laser pulses [45].

Despite being very promising, reports on optical excitation of spin dynamics in an opto-magnetic cavity are scarce [46]. The opto-magnonic cavity is demonstrated to provide a resonant dependency of IFE on the pump wavelength as well as a considerable increase in its magnitude at the wavelength of the cavity mode. At this, IFE effective magnetic field is localized within 30 nm along the film thickness. However, studies in [46] are limited by the spectral region of the cavity resonance and the structure contained only one magnetic layer forming the optical cavity. Here, we propose a concept aiming at extending spatial and spectral tunability of opto-magnetic excitation of spin waves. We tackle the problem by using MPC containing magnetic layers not only in the cavity but also in the Bragg mirrors. As a result, such MPC has several spectral features over a broad spectral range due to presence of cavity resonance as well as photonic bandgap edges.

In this article, we report on the optical pump-probe experiments with the one-dimensional MPC using femtosecond laser pulses to excite magnetization precession in the magnetic layers of the MPC. It is found that the amplitude of the excited spin precession is strongly dependent on the pump wavelength and, moreover, different layers of the MPC appear to be excited with different efficiency. We show that this results from the IFE in the MPC becoming spatially non-uniform with a character of its distribution tuned throughout the MPC by choosing a proper wavelength of the pump beam.

## 2 Sample and experimental setup

**Figure 1:**
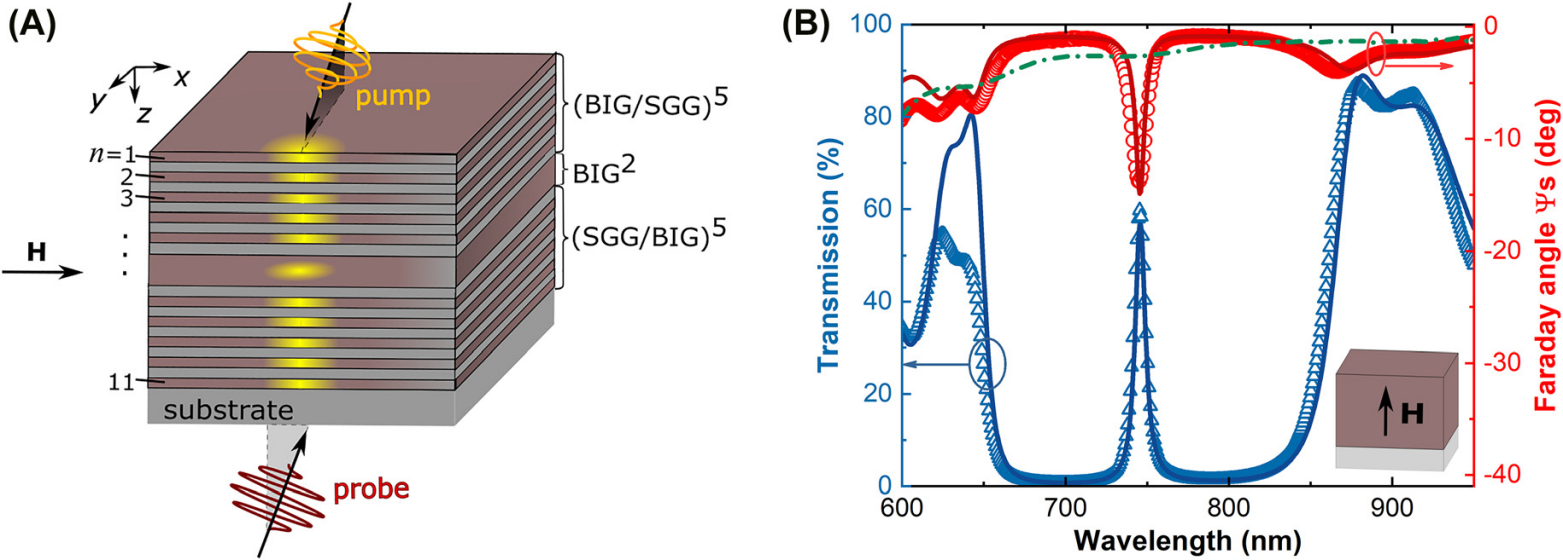
The schematic of the MPC sample and it spectra of transmittance and Faraday angle. (A) Scheme of the excitation and detection of magnetization precession in a magneto-photonic crystal. The magnetic film is 140 nm thick and is sandwiched between two magnetic Bragg mirrors composed of 5 pairs of magnetic dielectric layers BIG (70 nm, shown in brown) and nonmagnetic dielectric layers SGG (96 nm, shown in gray). The total thickness of the structure is 1.8 μm. The circularly polarized pump excites the structure, and the linearly polarized probe is used to observe the magnetization dynamics at variable time delays. The symbol *n* denotes the serial number of the magnetic layer in the structure. (B) Magneto-optical characterization of the MPC in the out-of-plane external magnetic field. Experimental (circles and triangles) and calculated (solid lines) optical transmittance and the Faraday rotation spectra for the TM-polarized light at the angle of incidence 17 deg and the MPC fully magnetized out-of-plane at 2.0 kOe (see inset). The measured Faraday rotation spectrum for a single 700 nm thick BIG film on the substrate is shown by the green dash-dot line.

The considered MPC structure consists of magnetic layers placed in the nanocavity region and in the Bragg mirrors (BMs) as well (Figure 1A) [47]. The sample is fabricated by subsequent RF-magnetron sputtering of different dielectric layers on Ca,Mg,Zr:Gd_3_Ga_5_O_12_(111) substrate. In particular, each BM is formed by five pairs of ferrimagnetic Bi_3_Fe_5_O_12_ (BIG) and diamagnetic Sm_3_Ga_5_O_12_ (SGG) layers. The iron garnet has a full substitution of rare Earth element sites by Bi ions which provides its high magneto-optical response. Thus, at a wavelength of *λ* = 750 nm its specific Faraday rotation is 3.59 deg/μm [47], whereas the conventional composition of Y_3_Fe_5_O_12_ rotates light polarization by 0.067 deg/μm only [48]. The cavity layer is also BIG. Therefore, the whole MPC sample has the following structure: substrate/back-BM/BIG/front-BM. The MPC is designed to obtain the optical cavity resonance at *λ*_r_ = 753 nm at normal light incidence. It is achieved by the layers’ thickness in the BMs corresponding to a quarter-wavelength: *h*_BIG(SGG)_ = *λ*_r_/4*n*_BIG(SGG)_, while the cavity layer thickness is half-wavelength: *h*_C-BIG_ = *λ*_r_/2*n*_BIG_. Refractive indices of BIG and SGG at *λ*_r_ are *n*_BIG_ = 2.679 and *n*_SGG_ = 1.960, respectively, which determines *h*_BIG(SGG)_ = 70 (96) nm and *h*_C-BIG_ = 140 nm. BIG saturation magnetization is *4πM* = 1400 G [47, 49] and the constant of uniaxial magnetic anisotropy is *K*_U_ = −8.66 × 10^4^ erg/cm^3^ (the anisotropy of the type “easy plane” type).

For a light incident, i.e., at 17 deg, the transmission spectrum of the MPC demonstrates a photonic bandgap it the spectral from 640 to 880 nm with a pronounced cavity mode positioned at 745 nm (Figure 1B, triangles). This corresponds to the tendency observed in [47] of a blue shift from resonance at normal incidence (753 nm). The Faraday rotation angle, Ψ_S_, has a maximum at the cavity resonance as well as at the edges of the photonic bandgap (Figure 1B, circles). The largest enhancement of the Faraday effect is achieved at the cavity resonance with factor of 5 with respect to a single magnetic film whose thickness 700 nm equals to the total thickness of the all magneto-active layers in the MPC (dashed line in Figure 1B). At the long-wavelength edge of photonic bandgap the enhancement level is also rather high, namely, a factor of 3. Since IFE is also determined by the optical field distribution within the magnetic layers, the spectral ranges of the cavity resonance and the one in the vicinity of the long-wavelength bandgap edge are particularly attractive for the opto-magnonic studies with fs-laser pulses.

For the optical excitation of spin dynamics in the MPC we use the optical pump-probe experimental technique (see Supplementary 1, Figure S1). The circularly polarized pump pulses with duration of 120 fs hit the sample to excite the magnetization precession via IFE. The precession is monitored by the variation of the Faraday rotation angle ψ of the linearly polarized probe pulse with duration of 120 fs transmitted through the sample at a time delay *t* with respect to the pump pulse. Modification of the excited spin dynamics is investigated at variation of pump’s central wavelength *λ*_pump_ from 732 to 910 nm. It covers most part of the photonic bandgap of the MPC including the cavity mode and bandgap’s long-wavelength edge (Figure 1B). The probe central wavelength *λ*_probe_ = 1050 nm is fixed in transmittance band of the MPC. The external magnetic field *H* = 1.05 kOe generated by an electromagnet is applied in the sample plane. All measurements are performed at room temperature with the pump fluence of 0.24 mJ/cm^2^ and incidence angles of the pump and probe of 17 deg.

## 3 Results and discussion

**Figure 2:**
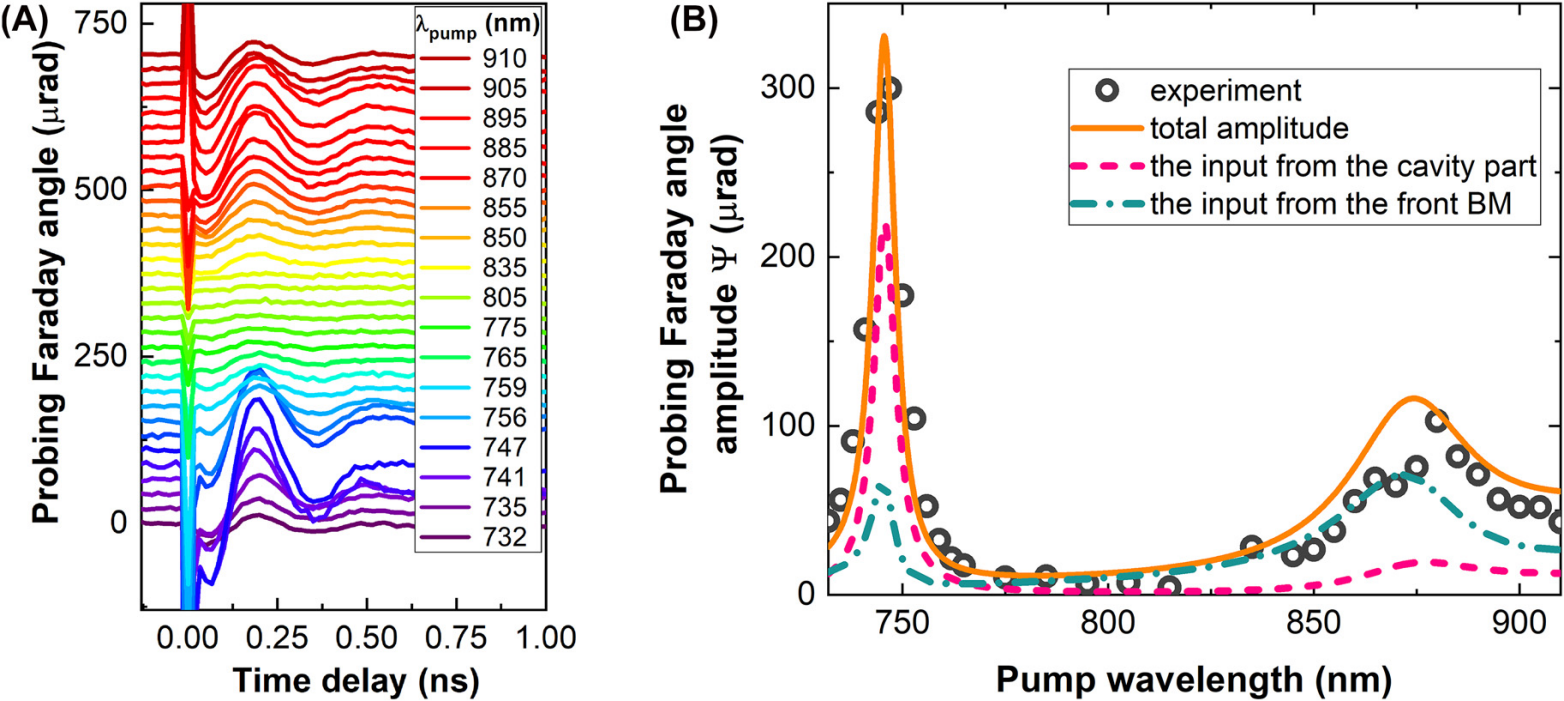
Probing Faraday angle. (A) Experimentally measured time-resolved change of the Faraday rotation of the probe pulses at different pump wavelengths from 732 to 910 nm. All curves have offsets for clarity of representation. (B) Experimentally measured (circles) and calculated (orange solid line) amplitudes of the oscillations seen in probe Faraday rotation as a function of the pump wavelength. The pink dashed curve represents the contribution of the nanocavity of the MPC and the blue dash-dotted curve provides the input from the front BM.

The signals ψ(*t*) at fixed *λ*_pump_ have an oscillatory behavior with decreasing amplitude, which corresponds to the decaying spin precession in BIG layers around the effective magnetic field ***H***_eff_ (Figure 2A). The precession results from illumination by a circularly polarized pump pulses with corresponding IFE as a triggering mechanism [1–3]. The oscillation can be approximated by a harmonic function with a decaying amplitude, $\psi \left(t\right)=\Psi \exp\left(-t/\tau \right)\sin\left(\omega t+\zeta \right)$ , where Ψ is an initial amplitude, *τ* is a decay time, *ζ* is an initial phase, and *ω* is the precession angular frequency. Dependence Ψ(*λ*_pump_) has a pronounced peak Ψ_cm_ at *λ*_pump_ = 745 nm coinciding with spectral position of the optical cavity mode resonance (Figure 2B). The second maximum of the amplitude, Ψ_bge_, appears at the bandgap edge, i.e., near *λ*_pump_ = 880 nm. The ratio of the two peaks is Ψ_cm_/Ψ_bge_ = 3.0. If *λ*_pump_ is inside the photonic bandgap, then the precession is hardly observed.

The IFE is described by the effective magnetic field ***H***_IFE_which depends on the optical field ***E*** of the light inside the magnetic material and the magneto-optical gyration parameter *g* [1]:

(1) $$H_{\text{IFE}}=-\frac{g}{16\pi M_{\text{s}}}Im\left(\left[E\times E^{*}\right]\right).$$

Let’s compare the ratio Ψ_cm_/Ψ_bge_ ∼ 3 with the ratios *T*_cm_/*T*_bge_ = 0.7, and Ψ_S cm_/Ψ_S bge_ ∼ 3.5 for transmittance and Faraday rotation of the MPC, respectively. It can be seen that the amplitude of spin dynamics is not directly governed by the value of transmitted light, while it is rather correlated to the Faraday rotation. The explanation is the fact that both the direct and the IFE in the MPC are governed by the optical field distribution inside the MPC layers [1, 50]. Indeed, the larger part of the optical energy is concentrated within the magnetic layers, the larger the Faraday rotation and *H*_IFE_ [Eq. (1)], with the latter resulting in higher efficiency of spin precession excitation.

**Figure 3:**
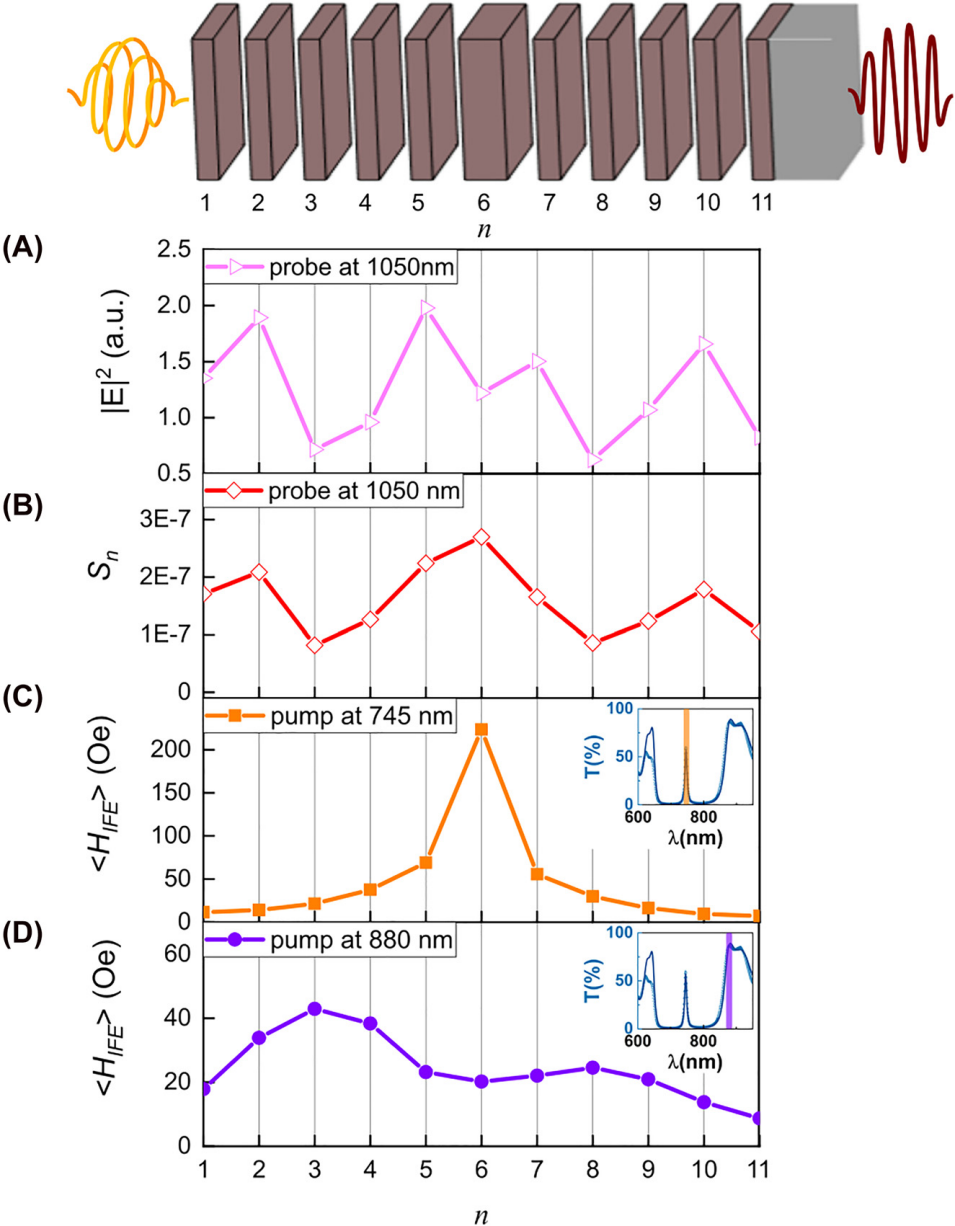
Calculated distribution of the electric field intensity, magneto-optical sensitivity and the averaged effective IFE field on the serial number of the magnetic layer in the structure. (A and B) Calculated distribution of the electric field intensity |*E*|^2^ (A) and magneto-optical sensitivity *S*_*n*_ (B) of the probe pulse across the structure. *n* denotes the index of the magnetic layer in the structure (see schematic representation on top). (C and D) Calculated distribution of the averaged effective IFE field inside the MPC at the pump wavelengths of 745 (C) and 880 nm (D). Insets in (C) and (D) show spectral position of the pump beam with respect to the MPC transmittance spectrum.

For the analysis of the experimental data, it is necessary to consider both the pumping and probing aspects. Let’s discuss the probing one at first. In fact, the excited spin dynamics is detected by the Faraday rotation of the polarization of the probe pulse, which field is distributed through the MPC non-uniformly (Figure 3A), as obtained from the transfer matrix calculations [51] (Supplementary). Thus, probe pulse has different magneto-optical sensitivity to the spins inside different MPC layers. The sensitivity to the *n*th layer, *S*_*n*_, can be defined as $S_{n}=\frac{\partial \Psi_{n}}{\partial \Theta_{n}}$ , where Ψ_*n*_ is the modulus of the Faraday rotation amplitude of the probe pulse caused by the spin precession in the *n*th magnetic layer of the MPC with an amplitude of Θ_*n*_ (Figure 3B). Since *S*_*n*_ depends on the distribution of the probe optical field inside the MPC, then it depends on the probe wavelength. In our experiments *λ*_probe_ is fixed at 1050 nm, and most of the probe energy is concentrated in magnetic layers with *n* = 2 and *n* = 5 of the front BM (Figure 3A). As a result, the highest value of *S*_*n*_ takes place for these layers (Figure 3B). Apart from that, the sensitivity is also high for the cavity layer (*n* = 6). It is due to its doubled thickness with respect to the other magnetic layers. At the same time, contribution of spin dynamics in some other layers (*n* = 3, 4, 7–11) to the probe Faraday rotation is lower since for those layers the magneto-optical sensitivity *S*_*n*_ is smaller. This peculiarity of the optical probing must be taken into account for the comparative analysis of the spin dynamics in different MPC layers.

As for the pumping, the distribution of the effective IFE field magnitude *H*_IFE_ [Eq. (1)] should be analyzed. Since the incidence angle is rather small and the refractive index of BIG is significant (*n*_BIG_ = 2.69 at *λ*_cm_ = 745 nm) the field *H*_IFE_ is directed almost normally to the layers’ surface inside the MPC. It affects the spins only during the pulse duration time, which is much smaller than the spin precession period. Therefore, medium magnetization is affected by the instant torque caused by IFE. Thus, magnetization dynamics can be considered as the decaying precession around the effective magnetic field ***H***_eff_ which is a sum of external and anisotropy fields. In the external magnetic field *H* = 1.05 kOe the sample is magnetically saturated in plane. As the iron garnet layers have easy-plane magnetic anisotropy, then at *H* = 1.05 kOe the effective magnetic field is directed parallel to the external field and equals to *H*_eff_ = *H* + 4*πM* − 2*K*_U_/*M*. Consequently, the precession of the magnetization in the *n*th layer is conveniently described by the temporal evolution of the angle *θ*_*n*_ between the magnetization and the layers’ plane (see Supplementary 1, Figure S1): $\theta_{n}\left(t\right)=\Theta_{n}\exp\left(-\frac{t}{\tau }\right)\sin\left(\omega t+\zeta \right)$ , where *τ* is a decay time, *ζ* is an initial phase and *ω* is frequency of precession. The initial amplitude Θ_*n*_ is proportional to *H*_IFE_ [52]:

(2) $$\Theta_{n}=\gamma {\left(1+\frac{4\pi M-\frac{2K_{\text{U}}}{M}}{H}\right)}^{-1/2}H_{\text{IFE},n}\Delta t,$$

where *γ* is a gyromagnetic ratio, and *H*_IFE,*n*_ is the IFE field within *n*th layer. It allows one to calculate amplitudes of the observed signals by $\Psi =\sum_{n=1}^{11}S_{n}\left\langle \Theta_{n}\right\rangle$ , where ⟨Θ_*n*_⟩ is the precession amplitude in the *n*th layer found from [Eq. (2)] and averaged over the layer thickness. We calculate *H*_IFE,*n*_ in each magnetic layer with the transfer matrix method. There is a good agreement between the simulated and experimental data (solid line and circles in Figure 2B), what confirms the adequacy of the used theoretical approach. Therefore, we employ it to investigate the distribution of the *H*_IFE_ inside the MPC.

The analysis shows that spin precession excited in the optical cavity and in the BMs gives different contributions to the observed signal depending on the excitation wavelength *λ*_pump_. Thus, the precession in the cavity mainly contributes to the signal at the cavity resonance (*λ*_pump_ = *λ*_cm_ = 745 nm) (dashed line in Figure 2B). On the over hand, the precession in the front BM dominates in the detected signal when the excitation is close to the edge of the photonic bandgap (*λ*_pump_ = *λ*_bge_ = 880 nm) (dash-dotted line in Figure 2B).

Let’s now consider *H*_IFE,*n*_ in different magnetic layers of the MPC at these pump wavelengths, *λ*_cm_ and *λ*_bge_ (Figure 3C, D). At the cavity resonance *λ*_cm_ most of the optical energy is distributed within the cavity BIG layer, *n* = 6 (Figure 3C), which leads to a relatively high average value of the effective IFE field in that layer, ${\left\langle H_{\text{IFE,}6}\right\rangle }_{\text{cm}}=$ 224 Oe, in comparison to the BIG layers of the BMs and to the single BIG film on an SGG substrate $\left\langle H_{\text{IFE}}^{\text{single}}\right\rangle =$ 15 Oe. Thus, for the cavity resonance, the magnetization dynamics is mostly excited in the central MPC layer, and the IFE field at the cavity resonance is approximately 15 times larger than for the single BIG film, which demonstrates a significant localization of the excitation within the cavity layer.

The situation becomes opposite when the excitation wavelength is tuned to the photonic bandgap edge. Optical pumping at *λ*_bge_ = 880 nm provides larger values of *H*_IFE,*n*_ in the magnetic layers of the BMs, while the cavity layer receives much smaller optical energy. For example, ${\left\langle H_{\text{IFE,}3}\right\rangle }_{\text{bge}}=$ 43 Oe, while ${\left\langle H_{\text{IFE,}6}\right\rangle }_{\text{bge}}=20$ Oe (Figure 3D). Contribution of the cavity layer (*n* = 6) to the probe Faraday rotation becomes much smaller than that of the front BM (compare dashed and dash-dotted lines at around 880 nm in Figure 2B) even in spite of the higher probing sensitivity to the cavity layer (*S*_6_ > *S*_*n*_ for *n* ≠ 6).

It should be noted, that in both cases the magnetic layers of the BMs are excited with different efficiency. Thus, at the cavity resonance, *H*_IFE_ is largest in the magnetic layers of the BMs close to cavity one (*n* = 5, 7) (Figure 3C), while at the bandgap edge *H*_IFE_ takes larger values in the middle of the front BM (*n* = 2, 3, 4). Therefore, by tuning *λ*_pump_, it is possible to modify the distribution of *H*_IFE_ across the MPC structure significantly and excite spin dynamics in the MPC layers with a good degree of spatial selectivity. For instance, relative excitation efficiency of the cavity layers with respect to the 3rd BM layer varies in the range from ${\left\langle H_{\text{IFE,}6}\right\rangle }_{\text{cm}}$ : ${\left\langle H_{\text{IFE,}3}\right\rangle }_{\text{cm}}$ = 10:1 to ${\left\langle H_{\text{IFE,}6}\right\rangle }_{\text{bge}}$ : ${\left\langle H_{\text{IFE,}3}\right\rangle }_{\text{bge}}$ = 1:2 as *λ*_pump_ is tuned from *λ*_cm_ to *λ*_bge_.

## 4 Conclusions

In this work, we demonstrate selective excitation and detection of the magnetization dynamics in magnetic layers of a one-dimensional MPC by femtosecond laser pulses. Results of optical pump-probe experiments demonstrate strong dependence of excited magnetization precession on the pump wavelength detuning with respect to the features of the MPC photonic bandgap.

The largest amplitudes are observed for pumping at the optical cavity resonance and at the edge of the photonic bandgap. Electric field of the circularly polarized pump pulse is distributed non-uniformly in the MPC, which results in concentration of spin angular momentum of light in some layers of the MPC and, therefore, enhancement of the optical impact on their magnetization and increase of the precession amplitude. In particular, for pumping at the cavity mode resonance the IFE field in the cavity layer is 15 times larger than in the single BIG film, which demonstrates a significant concentration of the optical field within the cavity layer.

Importantly, spin dynamics is excited in different magnetic layers of the structure with different efficiencies. Moreover, the pattern of the pump distribution inside the MPC depends strongly on its wavelength enabling to spatial tuning of the optical impact throughout the MPC. For example, if the pump wavelength is shifted from the cavity mode resonance to the long-wavelength edge of the photonic bandgap, then relative excitation efficiency of the cavity layer with respect to the layer of BM varies in the range from 10:1 to 1:2.

The concept of multilayered magneto-photonic structures supporting spatially and spectrally selective excitation and detection of magnetization dynamics is one of the cornerstones for the creation of 3D opto-magnonic components. A controllability of different magnetic layers by optical means shown in this work paves a way for denser magnon flow processing devices. Further research and engineering of a diverse MPC designs, involving operation in several bandgaps and cavity modes may open up new avenues for multilayered opto-magnonic systems.

## Supplementary Material

Supplementary Material Details
