# Supplementary material for: Spatially selective excitation of spin dynamics in magneto-photonic crystals by spectrally tunable ultrashort laser pulses
Source: Nanophotonics. 2022 May 31;11(13):3169–76. doi: 10.1515/nanoph-2022-0233 (PMC11501707; doi:10.1515/nanoph-2022-0233)
Supplement: Supplementary file 1 — Supplementary Material Details [file j_nanoph-2022-0233_suppl.docx]

**Spatially selective excitation of spin dynamics in magneto-photonic crystals by spectrally tunable ultrashort laser: supplemental document**

**1. Details of the pump-probe experiments**

| 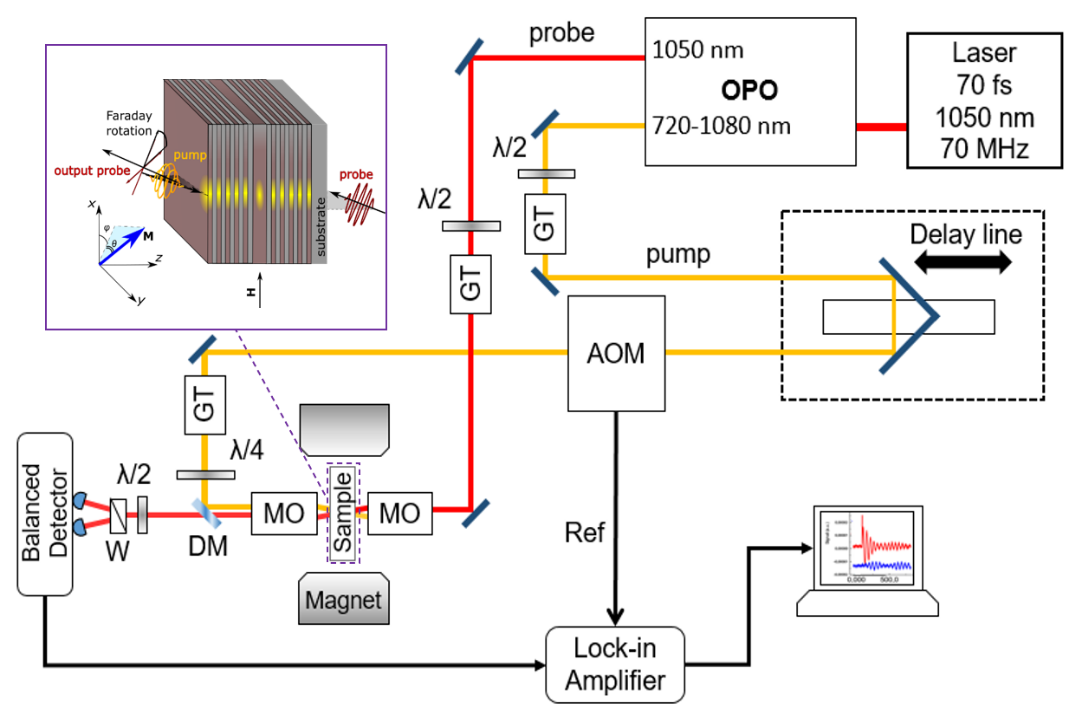 |
| --- |
| **Figure S1:** The experimental setup. OPO – optical parametric oscillator, AOM – acousto-optical modulator, GT – Glan-Taylor polarization prisms, MO – microobjectives, λ/2 and λ/4 – half-wave and quarter-wave plates respectively, W – Wollaston prism, DM – dichroic mirror. Inset: Scheme of the magnetic photonic crystal. The sample is a magneto-photonic microcavity formed by the magnetic film sandwiched in between two magnetic Bragg mirrors formed by several pairs of the dielectric layers N and M (gray – nonmagnetic layer, brown – magnetic layer). |

Experiments are performed using all-optical pump-probe technique with temporal resolution. The scheme of the setup and geometry of the experiments are shown in Supplement 1, Fig. S1. The magneto-photonic crystals sample is placed in a magnetic field of H = 1.05 kOe produced by the electromagnet and applied in the sample’s plane. The laser system of Yb-doped solid-state femtosecond oscillator TEMA-150 and the optical parametric oscillator TOPOL (both from Avesta-project, Russia) generates synchronized pump and probe 120-fs laser pulses with central wavelengths of 732-910 nm and 1050 nm, respectively. The pulses are focused on the opposite magneto-photonic crystals’s sides with reflective microscope objectives (15x, 0.4 NA, Newport, USA) into spots of 7 µm in diameter with oblique incidence of both pulses at 17 deg in orthogonal planes. The pump circular polarization is produced with achromatic quarter wave plate (AQWP05M-980, Thorlabs, USA).

The pump-induced dynamics of an out-of-plane component of the film magnetization is detected by measuring the magneto-optical Faraday rotation for the probe pulses using an optical bridge detector composed of a Wollaston prism and a balanced photodetector (PDB450C, Thorlabs, USA). The rotation is measured as a function of the time delay between the pump and probe pulses. The time delay is controlled with optomechanical delay line with retroreflector in pump path. The intensity of the pump pulses is modulated as a meander with an acousto-optic modulator at a frequency of 997 kHz. The frequency is used as a reference in lock-in amplifier, connected to balanced detector. The pump fluence is of 0.24 mJ/cm^2^; the probe fluence is about 20 times lower. The external magnetic field is 1.05 kOe. All measurements are performed at room temperature.

**2. Calculation of the Faraday effect and optical field distribution inside the magneto-photonic crystals**

The Faraday effect and optical field distribution in the magneto-photonic crystals are calculated by the transfer matrix method [1]. This method is based on the 4x4 matrix formalism, which operates with three main matrices: the column vector of four modal amplitudes; the dynamic matrix, which relates modal amplitudes and tangential components of the electric and magnetic fields; and the propagation matrix. The relation between the column vectors of the modal amplitudes before and after the multilayered structure allows the calculation of the reflection and transmission coefficients of light as well as the electromagnetic field distribution inside the structure.

**3. The enhancement factor of the inverse Faraday effect**

We considered MPCs with different number of layers in the Bragg mirrors and thus having different quality factors *Q* defined by the ratio of the cavity resonance wavelength, *λ_cm_*, to the resonance full-width at half maximum, *Δλ*: Q = *λ_cm_*/*Δλ.* It ranges from *Q* = 8 for the MPC with the Bragg mirrors containig only one pair of layers to *Q* = 287 for the MPC with 7 pair Bragg mirrors. The calculated distribution of the average effective field of IFE $H_{IFE}$ inside MPCs of different quality factors is shown in Fig. S2. The calculation was performed for the pump wavelength of the resonator mode *λ_cm_* and for the bandgap edge *λ_bge_* (Fig. S2).

On the basis of the IFE distribution in Fig. S2 we calculated the IFE enhancement factor (compared to a single film of the tickness equal to the total thickness of all magnetic layers in the MPC) versus the quality factor *Q* of the MPC (Fig. S3). For the experimentally studied MPC *Q* = 114. The enhancement factor was calculated for the cavity layer at *λ = λ_cm_* (black symbols) and for the central layer inside the front Bragg mirror at *λ = λ_bge_* (red symbols). It is seen that indeed, in both cases the field of the inverse Faraday effect increases with increase of the quality factor.

| 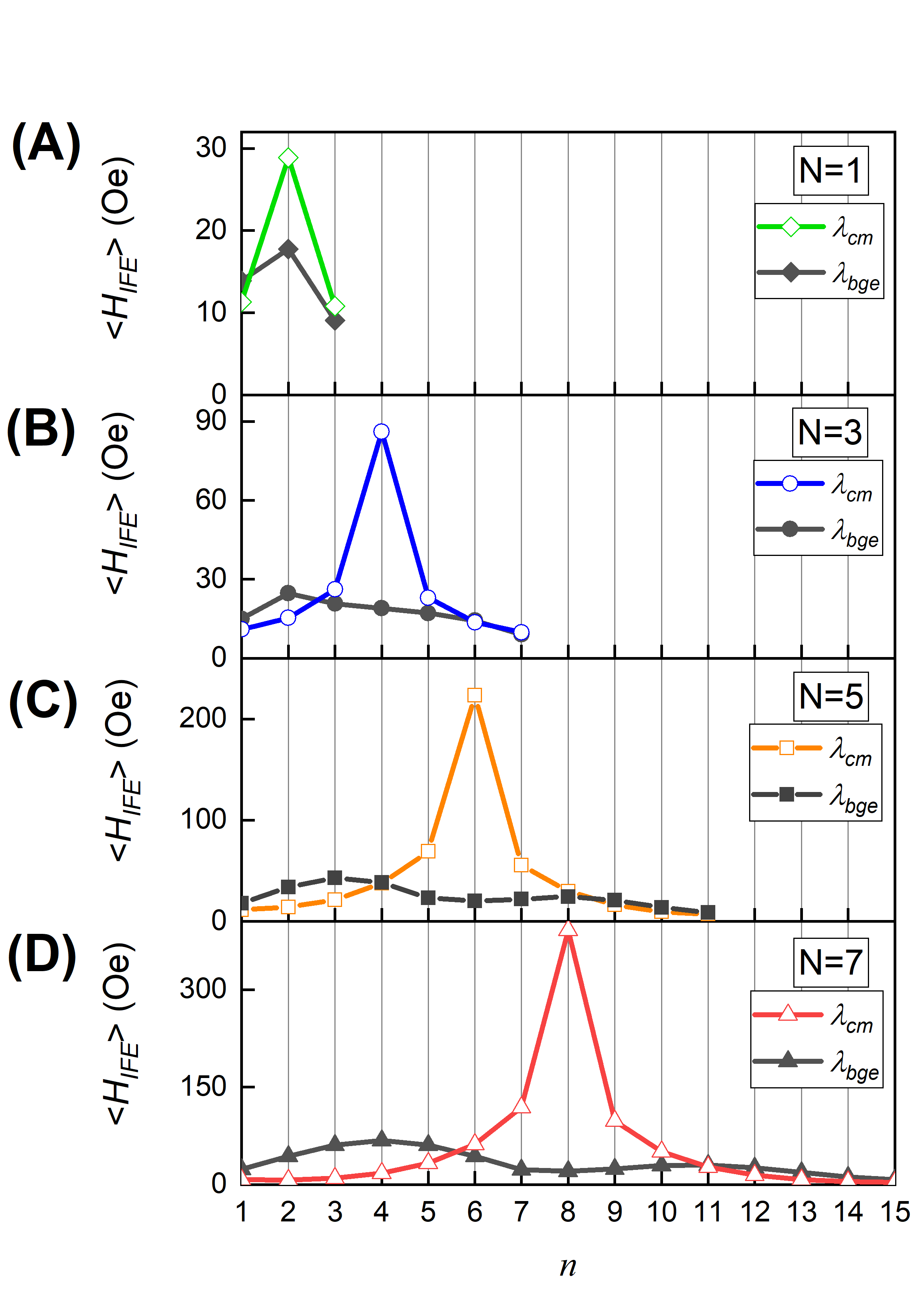 |
| --- |
| **Figure S2:** Calculated distribution of the average effective IFE field inside the MPC at the pump wavelengths of the cavity resonance and the edge of the photonic bandgap for (A)-1, (B)-3, (C)-5, (D)-7 pairs of the dielectric layers in the Bragg mirrors of the MPC. |

| 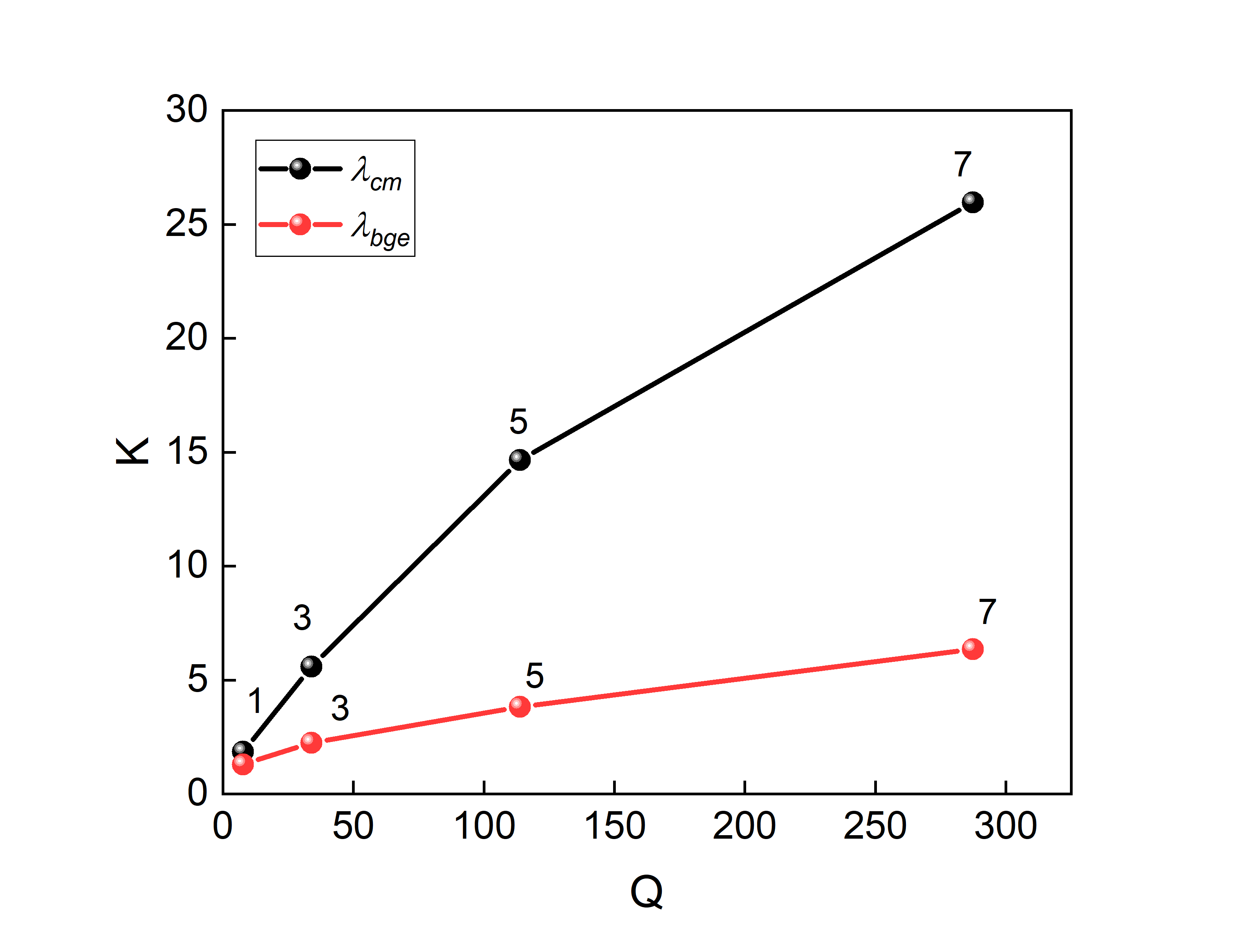 |
| --- |
| **Figure S3:** Enhancement factor of the inverse Faraday effect versus quality factor of the magnetophotonic crystals with different number of layers in the Bragg mirrors. Two cases are considered: the enhancement in the cavity layer at *λ = λ_cm_* (black symbols) and in the central layer inside the front Bragg mirror at *λ = λ_bge_* (red symbols). Numbers near the circles indicate number of the dielectric layer pairs in the Bragg mirrors of the MPC. |

References

1. A. Yariv, C.-S. Hong, and P. Yeh, "Electromagnetic propagation in periodic stratified media. I. General theory*," JOSA, Vol. 67, Issue 4, pp. 423-438 **67**, 423–438 (1977).
